# MicroRNA-27a Promotes Oxidative-Induced RPE Cell Death through Targeting FOXO1

**DOI:** 10.1155/2021/6666506

**Published:** 2021-11-01

**Authors:** Chengda Ren, Weinan Hu, Qingquan Wei, Wenting Cai, Huizi Jin, Donghui Yu, Chang Liu, Tianyi Shen, Meijiang Zhu, Xiuwei Liang, Jing Yu

**Affiliations:** ^1^Department of Ophthalmology, Shanghai Tenth People's Hospital, School of Medicine, Tongji University, Shanghai 200072, China; ^2^Department of Ophthalmology, Anhui University of Science and Technology, Huainan, Anhui 232001, China; ^3^Department of Ophthalmology, Jingdezhen No. 1 People's Hospital, Jingdezhen, Jiangxi 333000, China; ^4^Department of Ophthalmology, Ninghai First Hospital, Zhejiang Province 315600, China

## Abstract

Age-related macular degeneration (AMD) is a multifactor disease, which is primarily characterized by retinal pigment epithelium (RPE) cell loss. Since the retina is the most metabolically active tissue, RPE cells are exposed to consistent oxidative environment. So, oxidation-induced RPE cell death has long been considered a contributor to the onset of AMD. Here, we applied a retinal degeneration (RD) rat model induced by blue light-emitting diode (LED) and a cell model constructed by H_2_O_2_ stimulus to mimic the prooxidant environment of the retina. We detected that the expression of miR-27a was upregulated and the expression of FOXO1 was downregulated in both models. So, we furtherly investigated the role of miR-27a-FOXO1 axis in RPE in protesting against oxidants. Lentivirus-mediated RNA was injected intravitreally into rats to modulate the miR-27a-FOXO1 axis. Retinal function and histopathological changes were evaluated by electroretinography (ERG) analysis and hematoxylin and eosin (H&E) staining, respectively. Massive photoreceptor and RPE cell death were examined by terminal deoxynucleotidyl transferase-mediated dUTP nick end labeling (TUNEL). The damage to the retina was aggravated in the FOXO1 gene-knockdown and miR-27a-overexpression groups after exposure to LED but was alleviated in the FOXO1 gene-overexpression or miR-27a-knockdown groups. Dual luciferase assay was used to detect the binding site of miR-27a and FOXO1. Upregulated miR-27a inhibited the expression of FOXO1 by directly binding to the FOXO1 mRNA 3′UTR and decreased the autophagy activity of ARPE-19 cells, resulting in the accumulation of reactive oxygen species (ROS) and decrease of cell viability. The results suggest that miR-27a is a negative regulator of FOXO1. Also, our data emphasize the prominent role of miR-27a/FOXO1 axis in modulating ROS accumulation and cell death in RPE cell model under oxidative stress and influencing the retinal function in the LED-induced RD rat model.

## 1. Introduction

Age-related macular degeneration (AMD) is one of the principle causes of central vision decline and irreversible blindness in the elderly over 50 years old [1, 2]. It has been estimated that the number of patients diagnosed with AMD will reach 288 million by 2040 [3]. The most severe challenge to us is the lack of effective diagnostic strategies and interventions for early-stage AMD. Most patients are diagnosed at advanced stages and develop geographic atrophy (GA) or choroidal neovascularization (CNV) in the macula, leading to severe retinal pigment epithelial (RPE) cell and photoreceptor loss and irreversible blindness [4]. RPE plays a role as protector and scavenger to the retina. The main function of RPE is to maintain the blood-retinal barrier and phagocyte photoreceptor outer segment disks. In early AMD, RPE dysfunction is thought to be the primary change, after which progressive photoreceptor loss occurs. The health of RPE is of great importance to retinal function. Since the retina is a high oxygen demanding tissue, continuous exposure to light and strong oxygen metabolism force RPE to deal with a large amount of oxidative products, including reactive oxygen species (ROS) and lipofuscin [5]. Therefore, it is speculated that the aberrant antioxidant defense or the accumulation of oxidative damage may cause RPE dysfunction, furtherly inducing AMD [6, 7]. Thus, the establishment of an oxidative stress model is valuable to study the mechanism of AMD.

MicroRNAs (miRNAs) are a group of small noncoding RNAs (19-25 bp long) that regulate gene expression posttranscriptionally by binding to the 3′-untranslated regions (UTRs) of their target mRNA [8]. Studies have shown that miRNAs participate in the inflammatory response, oxidative stress, angiogenesis, and tumorigenesis [9, 10]. miRNAs also play a significant role in modulating biogenesis and differentiation of ocular cells. The preliminary study of our group suggested that miR-27a is upregulated in the blood of AMD patients compared with controls [11]. However, the questions of whether and how miR-27a takes part in AMD pathogenesis remained to be answered. Zhao et al. found that diabetes-induced oxidative stress increased miR-27a, and Deng et al. demonstrated that polycyclic aromatic hydrocarbon-associated oxidative stress induced the upregulation of miR-27a, in coke oven plants [12, 13]. Therefore, we hypothesize that miR-27a is an oxidation-related miRNA and can modulate RPE cell function under oxidative circumstance.

FOXO family member proteins, taking part in multiple intracellular signaling pathways, play an important role in regulating oxidative stress, autophagy, and apoptosis [14, 15]. Prooxidants have been demonstrated to activate the Akt-FOXO1 signaling pathway in RPE cells, which promotes Akt phosphorylation and reduces downstream FOXO1 expression [16]. Through prediction analysis via the TargetScan website, we proposed that the 3′UTR of FOXO1 may be a potential binding site of miR-27a. To verify the association between miR-27a and FOXO1 and their role in regulating RPE cell function, additional studies need to be performed.

In this work, we aimed at unveiling the role of miR-27a-FOXO1 axis in RPE cells under oxidative stress and in blue light-emitting diode- (LED-) induced retinal degeneration (RD) rat model. Our data emphasize the crucial role of ROS-miR-27a-FOXO1-autophagy feedback loop in regulating oxidative stress-induced cell death.

## 2. Materials and Methods

### 2.1. Experimental Animals

Male SD rats (age: 6–8 weeks old; weight: 180–240 g) were used for all relative experiments. All the animals were cared for by the Animal Experiment Center at Shanghai Tenth People's Hospital on the basis of the Association for Research in Vision and Ophthalmology (ARVO) Statement for the Use of Animals in Ophthalmic and Vision Research. All the rats were given free access to food and drinking water and were raised in a room with constant temperature (22°C). The facility is also equipped with an artificial 12 h light/dark cycle. All animal experiments were authorized by the institutional review board of Shanghai Tenth People's Hospital.

### 2.2. Blue LED Exposure

After 24 hours of dark adaptation, SD rats were topically treated with the eye drops of 0.5% tropicamide and 0.5% phenylephrine hydrochloride (Santen, Osaka, Japan). Wait for 30 minutes to ensure the pupil is sufficiently dilated. Next, the rats were given blue LED exposure (3000 lux; 460 ± 10 nm) for 2 hours (9:00 AM-11:00 AM) in a cage-like device. After the blue LED irradiation, the rats were displaced to a dark environment for 24 hours, then to the normal feeding environment.

### 2.3. Electroretinography

Electroretinography (ERG) responses assays were performed at 0, 3, 7, 14, and 28 days after light exposure. All the rats were dark adapted for 16 hours preliminarily. The manipulations were performed under a dim red light. The rats were anesthetized by an intraperitoneal injection of sodium pentobarbital (50 mg/kg) and an intramuscular injection of xylazine (6 mg/kg) before examination. Two gold wire ring contactors were put on the superficial corneal of both eyes, and the reference electrode was inserted into the middle of brow arch of the rat. The ground needle electrode was fixed subcutaneously at the rat's tail. Rats received a single light-flash stimulus (3000 cd/m^2^ for 10 ms). The amplitude of the a-wave is measured from the baseline to the bottom peak of the a-wave, and the amplitude of the b-wave is measured from the bottom peak of the a-wave to the apical point of the b-wave.

### 2.4. Hematoxylin and Eosin Staining

The rats were sacrificed after 0, 3, 7, 14, and 28 days of animal model establishment, and eyes were immersed in 4% paraformaldehyde for 24 h to be fixed. After fixation, paraffin-embedded serially sections of 3 *μ*m were cut carefully and then stained with hematoxylin-eosin (H&E). Photos were taken of the sections using a light microscope (Leica Microsystems, Wetzlar, Germany).

### 2.5. Immunohistochemistry

Immunohistochemical assay was conducted on the retinal tissue sections from the blue light-treated group and control group 0, 3, 7, 14, and 28 days after animal model establishment. The slides were dewaxed and rehydrated in the standard manner. 5% bicinchoninic acid (BCA) was used to block the slides (room temperature for 30 minutes). The sections were treated with the primary FOXO1 antibody (Cell Signaling Technology, Danvers, USA) (diluted 1 : 100 in blocking buffer), subsequently incubated overnight at 4°C. After incubation, the secondary antibody (1 : 200; Beyotime Institute of Biotechnology, Haimen, China) was used for incubation at 37°C for 30 minutes. Next, the sections were incubated with HRP-labeled avidin (Beyotime Institute of Biotechnology, Haimen, China) at 37°C for 30 minutes. At last, the sections should be treated with diaminobenzidine for visualization and with hematoxylin for counterstaining. The images were captured by a microscope (Leica Microsystems, Wetzlar, Germany).

### 2.6. Terminal Deoxynucleotidyl Transferase-Mediated dUTP Nick End Labeling

The rats were killed in 0, 3, 7, 14, and 28 days after animal model establishment, and histological sections were obtained. Apoptotic cell death was detected with terminal deoxynucleotidyl transferase-mediated dUTP nick end labeling (TUNEL) assay kit (Roche, Basel, Switzerland) according to the protocol provided by its manufacturer. The sections were treated with diaminobenzidine and then with hematoxylin. The sections were examined by a microscope equipped with epifluorescence (Leica, Heidelberg, Germany).

### 2.7. Intravitreal Administration in Rats

The rats were anesthetized with sodium pentobarbital (30 mg/kg; Shanghai Zangu Biotechnology Co., Ltd., Shanghai, China). Next, proparacaine hydrochloride eye drops (Alcon Ophthalmic Product Co., Ltd., TX, USA) were given to the experimental eyes for topical anesthesia. miR-27a and FOXO1 knockdown or overexpression was mediated by Lentivirus that was injected into the vitreous cavity of rats. The pleno-gph-FOXO1, which contains the rat FOXO1 RNA interference target GCAGCAGACACCTTGCTATTC, was integrated into the CMV-MCS-EF1*α*-GFP-t2a-PURO lentiviral vector (Zorinbio Biotech Company, Shanghai, China) between the BamhI and EcoRI restriction enzyme sites following the CMV promoter. The oligo sequences are 5′-GATCCGCAGCAGACACCTTGCTATTCCTTCCTGTCAGAGAATAGCAAGGTGTCTGCTGCTTTTTG-3′ (sense) and 5′-AATTCAAAAAGCAGCAGACACCTTGCTATTCTCTGACAGGAAGGAATAGCAAGGTGTCTGCTGC G-3′ (antisense). Using the above method, we synthetize another lentiviral vector named pleno-gph-rno-mir-27a-3p containing the rat rno-mir-27a-3p RNA interference target GTTCACAGCGGCTAAGTCCTGC. Its oligo sequences are 5′-GATCCGTTCACAGCGGCTAAGTCCTGCCTTCCTGTCAGAGCGGAACTTAGCCACTGTGAATTTTTG-3′ (sense) and 5′-AATTCAAAAAGTTCACAGCGGCTAAGTCCTGCTCTGACAGGAAGGCGGAACTTAGCCACTGTGAAG-3′ (antisense). The mock vector, which was synthesized without the target gene, was used as the negative control that is a blank virus. The lentiviral titer was 1 × 10^5^ infectious units per ml. During the operation, the action is as gentle as possible, avoiding lens damage and vitreoretinal injury.

### 2.8. RNA Isolation and Quantitative Real-Time Polymerase Chain Reaction

RNA was extracted from RPE samples of rats and ARPE-19 cells using TRIzol reagent (Invitrogen Corporation, CA, USA) based on the manufacturer's protocols. Complementary DNA was synthesized by 1 *μ*g of total RNA at 37°C for 15 minutes and 85°C for 5 seconds using a reverse transcription kit (Takara, Ohtsu, Shiga, Japan). The SYBR Green I reaction system kit (Toyobo CO., Ltd., Japan) was applied for quantitative real-time polymerase chain reaction (qRT-PCR). The expression of miR-27a and mRNA of FOXO1 was calculated by the 2^(-*ΔΔ*CT)^ method. Primers of miR-27a and U6 were obtained from Ribobio (Guangzhou, China). Other primers (Sangon Biotech, Shanghai, China) are as follows: human FOXO1 (forward: 5′-AGGGTTAGTGAGCAGGTTACAC-3′ and reverse: 5′-CTGCACACATTGGGCAAACA-3′); rat FOXO1 (forward: 5′-GGCGGGCTGGAAGAATTCAA-3′ and reverse: 5′-GAGCTGGTTCGAGGACGAAA-3′); human GAPDH (forward: 5′-GACTCATGACCACAGTCCATGC-3′ and reverse: 5′-AGAGGCAGGGATGATGTTCTG-3′); and rat GAPDH (forward: 5′-TTGTGCAGTGCCAGCCTC-3′ and reverse: 5′-GATGGTGATGGGTTTCCCGT-3′).

### 2.9. Western Blotting

The samples were lysed by RIPA lysis buffer (Beyotime Biotechnology, Nanjing, Jiangsu, China) which contains a protease inhibitor cocktail (EMD Millipore, Billerica, MA, USA) on ice for 30 minutes. Samples should be shaken slightly during lysis to ensure reacting sufficiently. All subsequent protein extracting procedures were performed in a standard manner. Then, standard electrophoresis procedure was performed and the proteins were transferred to nitrocellulose membranes, followed by blocking for 1 h. Subsequently, the membranes were treated with anti-FOXO1 antibody (1 : 1000; Cell Signaling Technology, Danvers, USA), *β*-actin antibody (1 : 1000; Cell Signaling Technology, Danvers, USA), and LC3B-I/II antibody (1 : 1000; Cell Signaling Technology, Danvers, USA) overnight at 4°C and then with secondary antibody (1 : 1000; Cell Signaling Technology, Danvers, USA) for 1 h at room temperature. The results were obtained by the Odyssey Infrared Laser Imaging system (LI-COR Corporate, NE, USA).

### 2.10. Culture of ARPE-19 Cells

The human retinal pigment epithelial cell line (ARPE-19) was obtained from the iCell Bioscience Company (Heidelberg, Australia). The cells were supplied with DMEM/F-12 (Thermo Fisher Scientific, Waltham, MA, USA) added with 10% fetal bovine serum (FBS), 100 U/ml penicillin, and 100 mg/ml streptomycin. The cells were cultured at 37°C under an atmosphere of 5% CO_2_. The ARPE-19 cell culture medium was changed every two days. The cell morphology was observed and photographed under an inverted microscope.

### 2.11. Cell Viability Assay

ARPE-19 cells were seeded in a 96-well plate (Corning Incorporated, NY, USA; 30,000 cells/cm^2^) and then were treated with 0, 100, 200, 400, and 800 *μ*M of H_2_O_2_ for 0.5 h, 1 h, 1.5 h, 2 h, 2.5 h, and 3 h, respectively. After stimulation at the indicated times, 10 *μ*l of CCK-8 solution (Yeasen, Shanghai, China) was added to each well to detect cell viability. The cell viability was denoted by the absorption at 450 nm wavelength, which was detected with a microplate reader (Synergy H4; BioTek, Winooski, VT, USA).

### 2.12. Transfection Assays

Stably growing ARPE-19 cells were seeded in a 6-well plate (Corning Incorporated, NY, USA) at 10 × 10^4^ cells/well. When the cells met the confluence between 30% and 50% within 24 h, the culture medium was discarded and PBS was used for washing. Next, 1 ml of Opti-MEM medium containing the lipo2000-miRNA mimic mixture was applied in transfection. After 6 h's contacting, the Opti-MEM medium was discarded, the cells were washed with PBS, and then, 2 ml of fresh F12 complete medium was added to the culture well. After 48 hours, the transfection efficiency was monitored by qRT-PCR.

### 2.13. Dual Luciferase Reporter Gene

The psiCHECK vector (Promega, Madison, USA) was constructed from wild-type 3′-UTR of FOXO1 or mutant 3′-UTR of FOXO1, respectively. The cells were plated into 48-well plates (Corning Incorporated, NY, USA) and grew to 80% confluence within 12 h. Then, plasmid and miRNA oligo were cotransfected into cells. Firefly luciferase (FL) and Renilla luciferase (RL) intensities were monitored after 24 h according to the manufacturer's protocol (Promega, Madison, USA). The RL intensities were normalized to FL intensities and denoted by fold change relative to the control group.

### 2.14. Measurement of Intracellular ROS

ARPE-19 cells with stable growth were plated into confocal microscope dishes at 4.0∗10^4^ cells per well. When the cells reached to 50-70% confluence, they were pretreated with 200 nM rapamycin or 1 mM 3-MA for 2 h. Then, H_2_O_2_ was added for 0.5 h. Next, 1 *μ*l of a H2DCFDA probe (Sigma-Aldrich, St Louis, USA) was added to the dishes. Photos were taken by a confocal microscope every minute. After that, we used flow cytometry to quantify the production of ROS in cells with different conditions as indicated in results and the H2DCFDA probe was used following the manufacturer's instruction.

### 2.15. Statistical Analysis

All data in this study were represented as means ± standard deviation (SD). One-way analysis of variance (ANOVA) or unpaired *t*-tests (two tailed) were performed appropriately to evaluate the significance of difference between two groups. Statistical graphs were produced by GraphPad Prism version 6.0 (GraphPad, USA). Statistical analyses were conducted with GraphPad Prism or Statistical Product and Service Solutions 23.0 (SPSS 23.0) software (IBM, Armonk, NY, USA). Values of *P* < 0.05 were considered to be statistically significant.

## 3. Results

### 3.1. Blue LED Exposure Induces Retinal Degeneration (RD)

**Figure 1:**
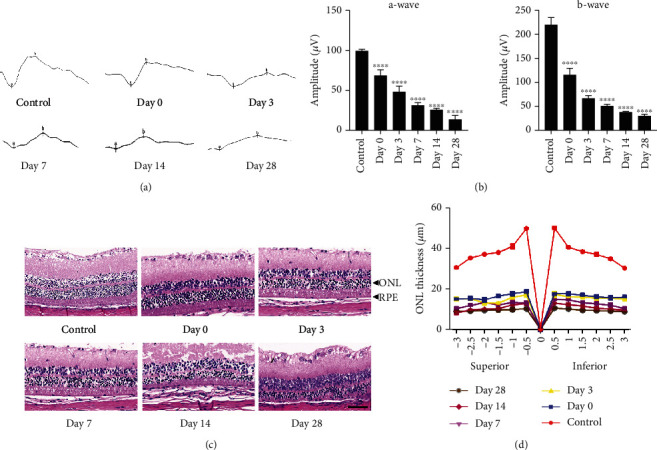
Functional and histological assessment of the RD rat model. (a) Representative ERG response graph of the control group and RD model group at the indicated times. All ERG waves gradually decreased with time. (b) ERG amplitudes of the a-waves and b-waves. (c) Representative H&E staining of retinal sections from the control group and RD rat model group at the indicated time points. There is an obvious disorder of retinal structure and a decrease of retinal thickness over time (scale bar = 50 *μ*M). (d) Statistical analysis of the ONL thickness in the above groups (superior to inferior from the optic nerve head). The data are calculated as means ± SD. ^∗^*P* < 0.05, ^∗∗^*P* < 0.01, and ^∗∗∗∗^*P* < 0.0001, comparisons versus control, *n* = 6 (eye samples in each group).

Here, we used blue LED to induce the retinal degeneration model in rats and detected the expression of miR-27a and FOXO1 in that model. First, we evaluated whether the RD rat model was successfully established. ERG recordings were performed at 0 h, 3 d, 7 d, 14 d, and 28 d after blue LED exposure to evaluate the physiology function of the retina. The amplitudes of the a-waves and b-waves of all blue LED-exposed animals were markedly decreased compared with those of the control group. Moreover, the amplitudes showed progressive reduction in a time-dependent manner (Figures 1(a) and 1(b)). To investigate the influences of blue LED emissions on retinal morphology, H&E staining was conducted at the time points of 0 h, 3 d, 7 d, 14 d, and 28 d after blue LED stimuli. The blue LED-exposed group exhibited significant retinal structure disorder, massive photoreceptor loss, and significant decline of the thickness of ONL compared with the control group (Figures 1(c) and 1(d)). Our results demonstrated that blue LED successfully induced retinal degeneration in rats and the characteristics of this model (RPE cells and photoreceptors loss) can mimic the feature of AMD in some degree.

### 3.2. Expression and Effects of miR-27a-FOXO1 Axis in the RD Rat Model

**Figure 2:**
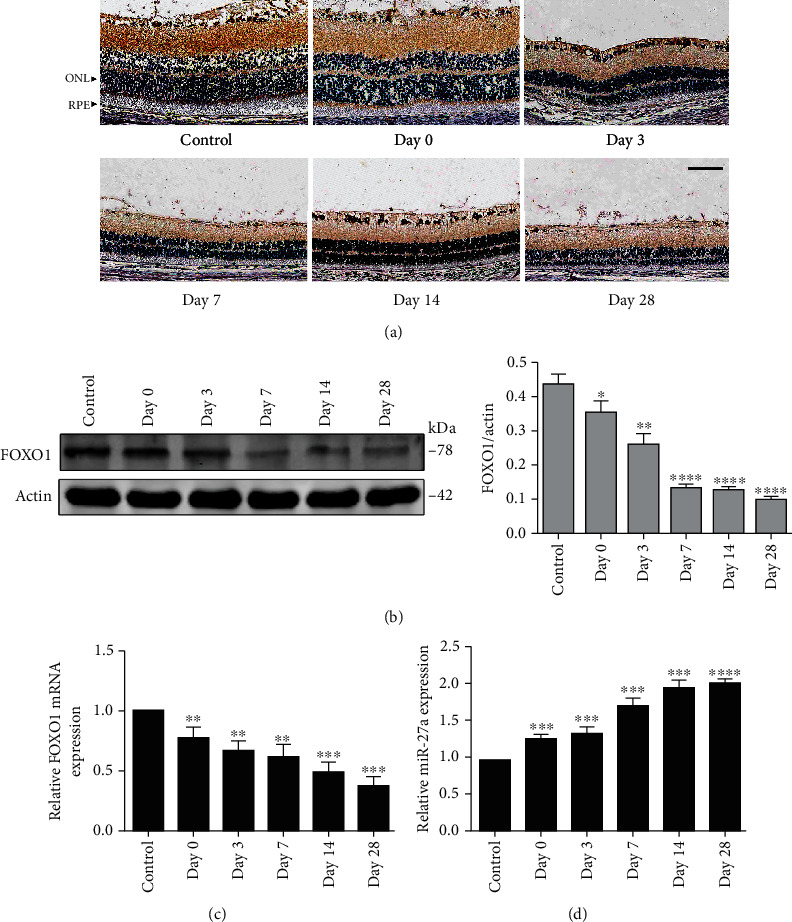
FOXO1 and miRNA-27a expression after blue LED exposure *in vivo*. (a) The FOXO1 protein levels were examined by immunohistochemistry after blue LED exposure at the indicated times (scale bar = 50 *μ*M). (b) The western blot analysis and the statistical results of FOXO1 protein levels in the RPE-choroid-sclera complex at the indicated times after blue LED exposure. (c, d) The FOXO1 mRNA levels and miRNA-27a expression levels of the RPE-choroid-sclera complex were detected by qRT-PCR at the indicated times after blue LED exposure; 2(-*ΔΔ*CT) method was used for quantification analysis. The data are expressed as means ± SD. ^∗^*P* < 0.05, ^∗∗^*P* < 0.01, ^∗∗∗^*P* < 0.001, and ^∗∗∗∗^*P* < 0.0001, comparisons versus control, *n* = 6 (eye samples in each group).

As we previously showed that miR-27a is upregulated in the peripheral blood of AMD patients, we detected the expression levels of miR-27a and its predicted target gene FOXO1 in retinal tissues by qRT-PCR, immunohistochemistry, and western blotting. The results indicated that the expression levels of miR-27a were increased with the exposure time of blue LED while the FOXO1 mRNA and protein levels showed an inverse tendency (Figures 2(a)–2(d)).

**Figure 3:**
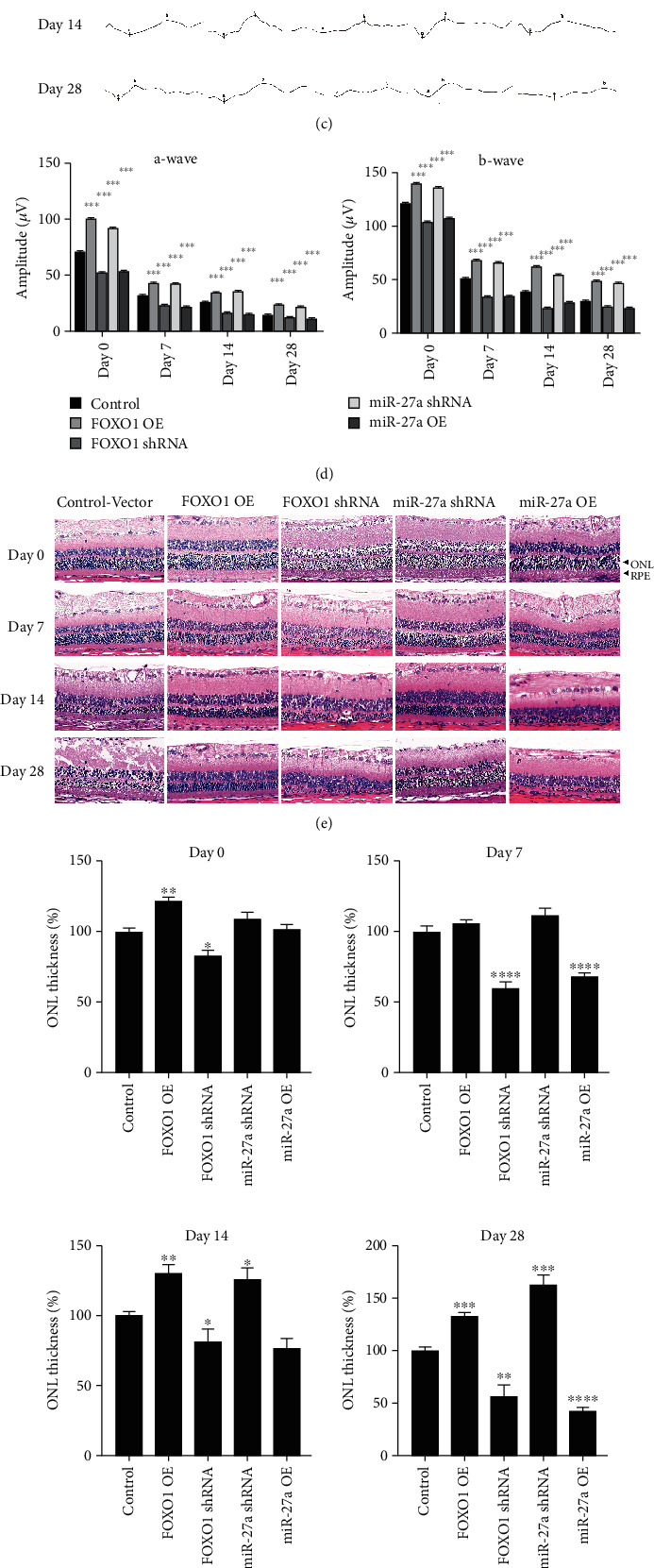
The morphological and functional changes after modulating miR-27a-FOXO1 axis in the RD rat model. (a) The relative expression level of miR-27a of rats treated with miR-27a OE (miR-27a overexpression) and miR-27a shRNA. (b) The relative expression level of FOXO1 mRNA of rats treated with FOXO1 OE (FOXO1 overexpression) and FOXO1 shRNA. (c, d) The representative images and mean amplitudes of ERG responses in 3000-lux blue LED-exposed retinas of the control blank and experimental Lentivirus-mediated injection groups at different time points (e, f) H&E staining of retinal sections and the ONL thickness measurement of the control blank Lentivirus and experimental Lentivirus-mediated injection groups at different time points after blue LED exposure (scale bar = 50 *μ*M). (c) TUNEL of representative sections from the control blank Lentivirus and experimental Lentivirus-mediated injection groups at different time points (Scale bar =50 *μ*M). The data are analyzed as means ± SD. ^∗^*P* < 0.05, ^∗∗^*P* < 0.01, and ^∗∗∗^*P* < 0.001, comparisons versus control, *n* = 6 (eye samples in each group).

Thus, we hypothesized that the miR-27a-FOXO1 axis might participate in the onset of retinal degeneration. To clarify the exact role of this axis, we used lentiviral to modulate the expression of FOXO1 and miR-27a in the retina of rats. We first verified the effectiveness of the lentiviral (Figures 3(a) and 3(b)). After that, ERG, H&E staining, and TUNEL assays were performed to monitor the physiology, morphology, and apoptosis of the retinal and RPE (cells) in rats. As shown in the ERG assay at time points of 0 h, 7 d, 14 d, and 28 d after blue LED exposure, there was a more severe functional damage in the FOXO1-knockdown group and miR-27a-overexpression group. Conversely, we observed a dramatic increase in the mean amplitudes of a- and b-waves in the FOXO1-overexpression group and miR-27a-knockdown group (Figures 3(c) and 3(d)). The results indicated that the abnormal miR-27a/FOXO1 expression may aggravate retinal damage. Consistent with the ERG assay, more severe disruption of the retinal morphology was detected in the FOXO1-knockdown group and miR-27a-overexpression group. As expected, the FOXO1-overexpression group and miR-27a-knockdown group demonstrated slighter disruption of the retinal structure as reflected by the more regular RPE layer, the less photoreceptor loss, and the maintenance of ONL thickness (Figures 3(e) and 3(f)). Furthermore, we conducted TUNEL analysis to assess the apoptotic rate of retinal cells.  Figure 3(g) shows that both the FOXO1-knockdown group and miR-27a-overexpression group had more retinal cells death (both RPE cells and photoreceptors) after blue LED exposure. Meanwhile, the apoptotic rate was decreased in the FOXO1-overexpression group and miR-27a-knockdown group. Our results demonstrated that blue LED enhanced miR-27a expression and suppressed the expression of FOXO1. Additionally, overexpression of miR-27a and downregulation of FOXO1 led to more severe damage both in retinal function and structure after blue LED exposure.

### 3.3. H_2_O_2_ Induces Abnormal Expression of miR-27a-FOXO1 Axis in ARPE-19 Cells

**Figure 4:**
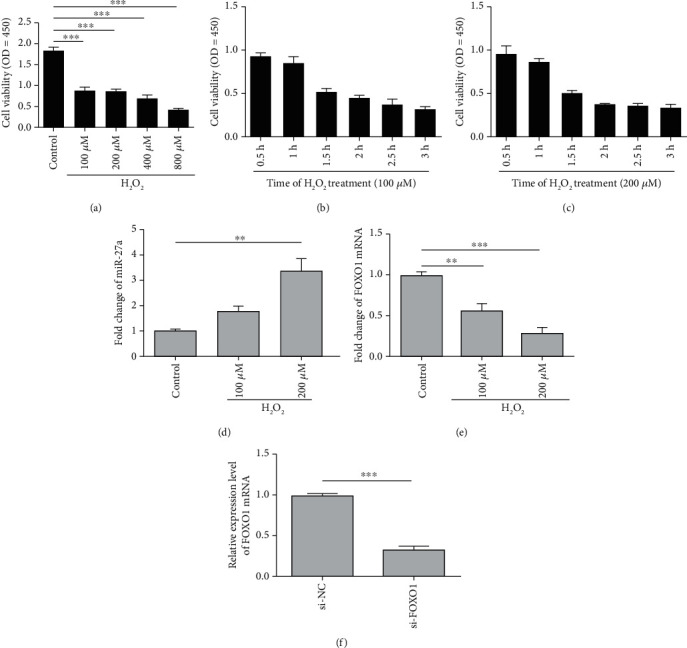
Establishment of the oxidative stress response model *in vitro* in ARPE-19 cells. (a) ARPE-19 cells were treated with 100-800 *μ*M H_2_O_2_ for 0.5 h, and cell viability was measured at a wavelength of OD = 450 by CCK-8 assay. (b, c) ARPE-19 cells were under 100 and 200 *μ*M H_2_O_2_ treatment for 0.5–3 h, and cell viability was measured by CCK-8 assay. (d, e) Quantitative real-time PCR results of the relative miRNA-27a and FOXO1 mRNA expression level of ARPE-19 cells under H_2_O_2_ treatment; 2(-*ΔΔ*CT) method was used for quantification analysis. (f) Quantitative real-time PCR was applied to analyze the FOXO1 mRNA expression in ARPE-19 cells transfected with the si-FOXO1 compared with the NC group; 2(-*ΔΔ*CT) method was used for quantification analysis. The data are expressed as means ± SD. ^∗∗^*P* < 0.01 and ^∗∗∗^*P* < 0.001. All experiments were repeated three times independently.

Because RPE is the important sustainer of outer retinal function, the pathological changes of outer retina are frequently secondary to the damage of RPE. Thus, we speculate that dysfunction of RPE cells plays a significant role in the miR-27a-related oxidative retinal injury. We treated ARPE-19 cells with different concentrations of H_2_O_2_ for 0.5 h (Figure 4(a)) and with 100 *μ*M or 200 *μ*M H_2_O_2_ for 0.5 h to 3 h (Figures 4(b) and 4(c)) to establish the oxidative cell model. ARPE-19 cells manifested a decline in cell viability when they were stimulated with H_2_O_2_. To mimic the oxidative environment and retain cell function as more as possible, we chose 100 *μ*M H_2_O_2_ for 0.5 h to induce the oxidative cell model. Next, we used 100 *μ*M and 200 *μ*M H_2_O_2_ to treat ARPE-19 cells for 0.5 hours and found that the expression of miR-27a was upregulated, while the expression of FOXO1 mRNA was downregulated in a dose-dependent manner (Figures 4(d) and 4(e)). The results demonstrated a similar tendency of miR-27a-FOXO1 axis in the oxidative cell model with that in our rat model. Moreover, FOXO1 mRNA level was significantly downregulated when ARPE-19 cells were transfected with si-FOXO1 for 48 h (Figure 4(f)).

**Figure 5:**
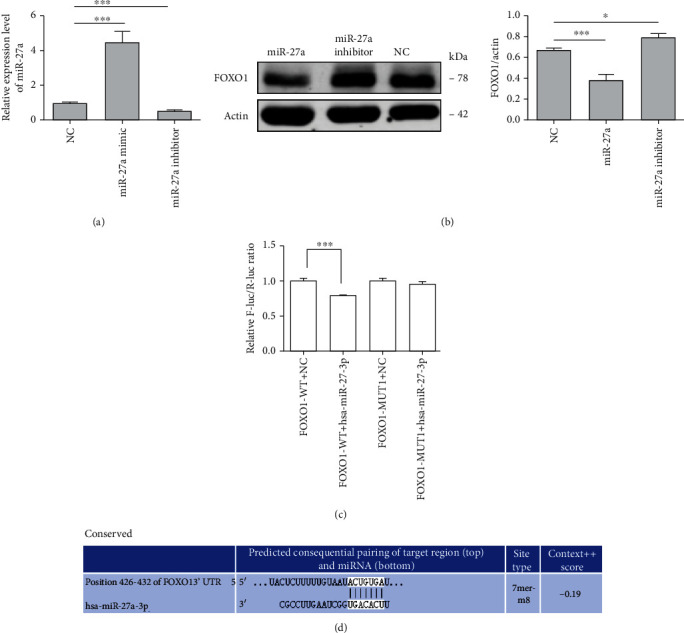
miRNA-27a regulates FOXO1 expression through binding to the 3′-UTR region. (a) ARPE-19 cells were transfected with negative control, miRNA-27a mimic, and miRNA-27a inhibitor for 48 hrs, and qRT-PCR was applied to show the relative abundance of miRNA-27a in ARPE-19 cells. U6 was applied as an internal control. (b) Negative control, miRNA-27a mimic, and miRNA-27a inhibitor were transfected into ARPE-19 cells for 48 hrs, and the FOXO1 protein levels were measured by western blotting. Actin was used as an internal control. (c) The dual luciferase assay verified the binding site of miRNA-27a to the FOXO1 3′-UTR. ARPE-19 cells were transfected with wild-type FOXO1 or mutant-type FOXO1 with negative control and miRNA-27a mimic, respectively, for 48 hrs, and the dual luciferase activity was measured. (d) The 3′-untranslated region of FOXO1 mRNA targeted by miRNA-27a was predicated through the TargetScan website. The data are expressed as means ± SD. ^∗^*P* < 0.05 and ^∗∗∗^*P* < 0.001. All experiments were repeated three times independently.

Western blotting and qRT-PCR were performed to evaluate whether miR-27a can modulate FOXO1 expression in ARPE-19 cells. As shown in Figure 5(a), transfection of the miR-27a mimic significantly upregulated the expression of miR-27a while transfection of the miR-27a inhibitor repressed the expression of miR-27a slightly. Furtherly, we detected the FOXO1 protein level in cells transfected with miR-27a, miR-27a inhibitor, and miR-NC. Results showed that overexpression of miR-27a is correlated with suppression of FOXO1 (Figure 5(b)). To further investigate whether miR-27a exerts its function by binding to the 3′-UTR region of FOXO1 mRNA, we conducted luciferase report assay. As shown in Figures 5(c) and 5(d), luciferase activity was repressed significantly in miR-27a overexpressed cells. Then, we detected a diminished luciferase intensity when we generated a mutation at the binding site of miR-27a and FOXO1 mRNA and treated the mutated cells with miR-27a mimic. Therefore, these results together allowed us to conclude that H_2_O_2_ treatment of ARPE-19 cells promotes miR-27a expression and miR-27a targets the 3′-UTR of FOXO1 mRNA and then inhibits its gene expression.

### 3.4. miR-27a-FOXO1 Axis Regulates Cell Viability through Inhibiting Autophagy and Antioxidation

**Figure 6:**
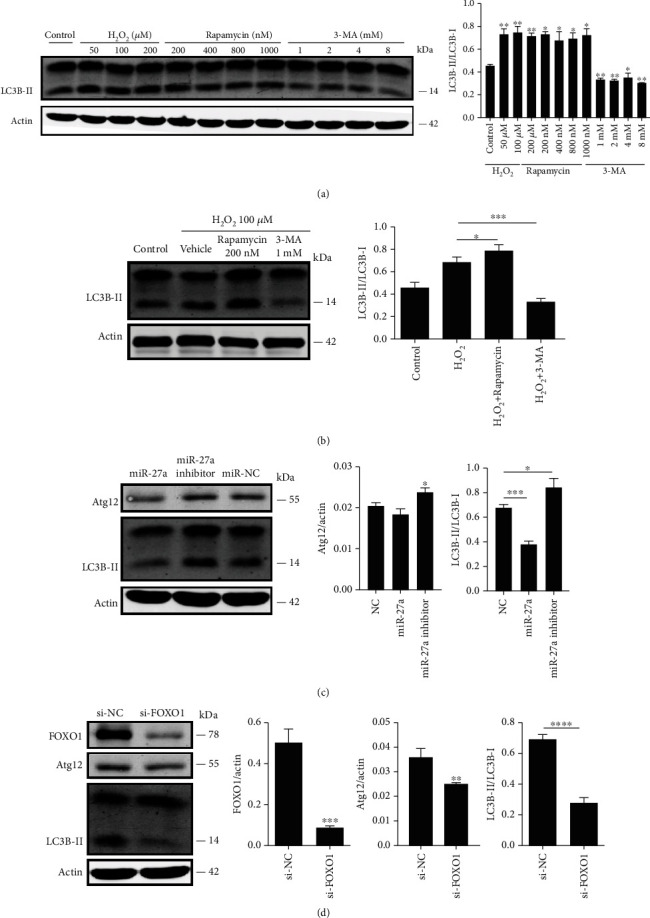
The miRNA-27a-FOXO1 axis regulates ARPE-19 cell autophagic activity. (a) ARPE-19 cells were treated with different concentrations of H_2_O_2_, 3-MA, or rapamycin, and the expression of autophagic protein LC3B-II was detected by western blotting. Actin was used as an internal control. (b) Cells were pretreated with vehicle, rapamycin (200 nM), or 3-MA (1 mM), respectively, and then challenged with H_2_O_2_ (100 *μ*M). LC3B-II protein levels were detected by western blot assay. (c) Western blot results of ARPE-19 cells transfected with miR-27a, miR-27a inhibitor, or miR-NC. (d) ARPE-19 cells were transfected with si-NC or FOXO1 siRNA for 48 hrs, and the expression levels of FOXO1, Atg12, and LC3B-II were examined by western blotting. Actin was used as an internal control. The data are shown as means ± SD. ^∗^*P* < 0.05, ^∗∗^*P* < 0.01, ^∗∗∗^*P* < 0.001, and ^∗∗∗∗^*P* < 0.0001. All experiments were repeated three times independently.

The preceding results suggested that abnormal miR-27a-FOXO1 axis induces functional and structure damage of the rat retina. FOXO1 participates in various cell functions including autophagy, antioxidation, and apoptosis through modulating the transcription of its downstream genes [17]. Thus, to understand the specific mechanism in our oxidative cell model, we transfected the miR-27a mimic, miR-27a inhibitor, and si-FOXO1 into ARPE-19 cells and monitored changes in the cell viability, autophagy, and ROS levels. First, we detected the autophagy level of ARPE-19 cells when treated with H_2_O_2_ independently or with rapamycin and 3-MA, respectively.  Figure 6(a) showed that H_2_O_2_ could enhance autophagy level of ARPE-19 cells. Treatment with rapamycin also enhanced autophagy level while 3-MA significantly inhibited cell autophagy activity. Then, we pretreated cells with 200 nM rapamycin or 1 mM 3-MA and then challenged with 100 *μ*M H_2_O_2_. Results demonstrated that rapamycin could furtherly enhance the autophagy activity of cells exposed to H_2_O_2_ while 3-MA showed a reverse tendency (Figure 6(b)). After that, we questioned whether miR-27a-FOXO1 axis can modulate autophagy in ARPE-19 cells. Western blot analysis showed that the ratio of LC3B-II/LC3B-I was dramatically decreased in the oxidative cells transfected with the miR-27a mimic and si-FOXO1, while an opposite response was observed for cells transfected with the miR-27a inhibitor (Figures 6(c) and 6(d)). Atg12, a downstream autophagic-related gene of FOXO1, was also repressed when cells were treated with miR-27a mimics and si-FOXO1. This may partially account for the correlation of expression of FOXO1 and autophagic activity in ARPE-19 cells. Our results demonstrated that the overexpression of miR-27a inhibited autophagy level through targeting FOXO1 and inhibiting expression of Atg12 in ARPE-19 cells.

**Figure 7:**
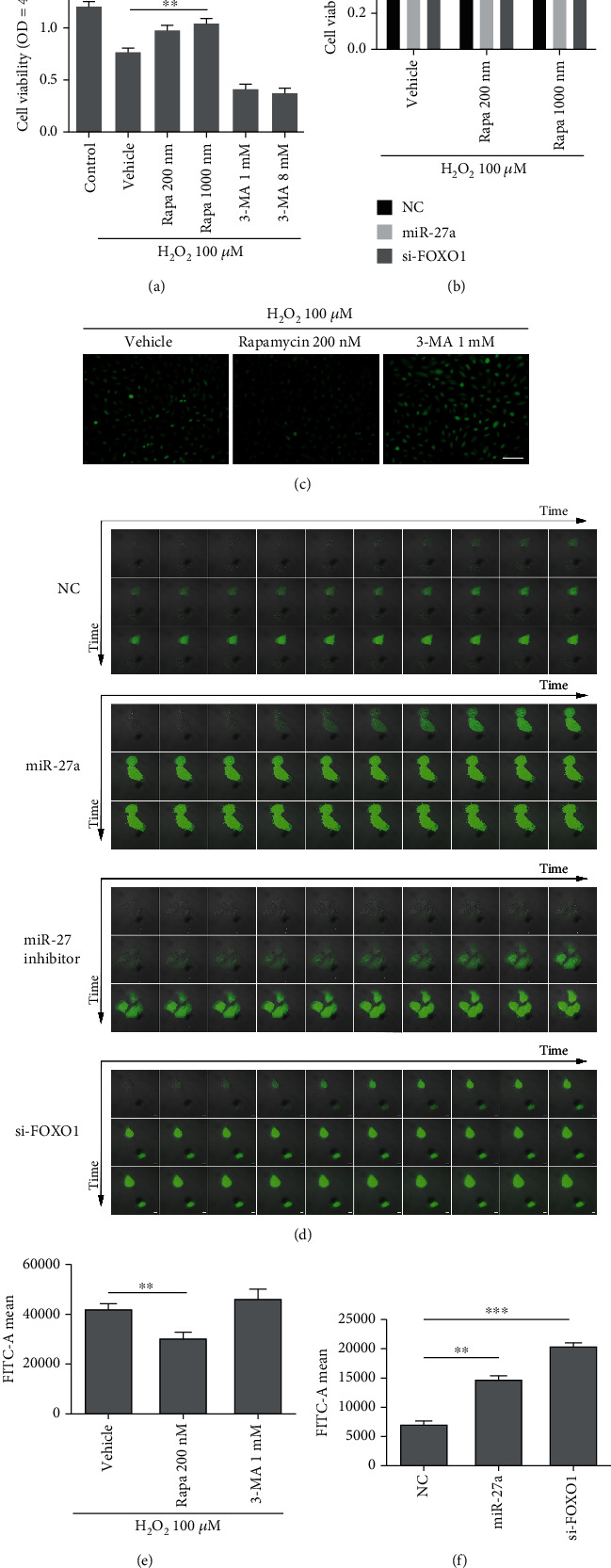
The perturbation of miRNA-27a-FOXO1 axis reduces antioxidative ability of ARPE-19 cell in an autophagy-dependent way. (a) ARPE-19 cells were pretreated with the indicated concentrations of rapamycin and 3-MA for 2 hrs and were stimulated with H_2_O_2_ (100 *μ*M), and then, the cell viability was measured by CCK-8 assay. (b) ARPE-19 cells were transfected with the negative control, miRNA-27a, and FOXO1 siRNAs for 48 hrs and then were pretreated with the indicated concentrations of rapamycin for 2 hrs. Thereafter, the cells were stimulated with H_2_O_2_ (100 *μ*M) and the cell viability was measured by CCK-8 assay at 450 nm wavelength. (c) H2DCFDA staining of oxidative APRE-19 cells in the indicated groups (scale bar = 100 *μ*M). (d) The fluorescence of intracellular ROS in the indicated groups was measured in a time-dependent manner (starting time = 0 min; time interval = 1 min; scale bar = 10 *μ*M). Intracellular ROS of ARPE-19 cells stimulated with 100 *μ*M H_2_O_2_, H_2_O_2_ + 200 nM RAPA, and H_2_O_2_ + 1 mM 3-MA were subjected to flow cytometry analysis. (f) ARPE-19 cells were transfected with the negative control, miRNA-27a, and FOXO1 siRNA, and the intracellular ROS was analyzed by flow cytometry. Rapa: rapamycin. The data are shown as means ± SD. ^∗^*P* < 0.05, ^∗∗^*P* < 0.01, and ^∗∗∗^*P* < 0.001. The experiments were repeated three times independently.

Next, the CCK-8 assay was conducted to evaluate whether miR-27a-FOXO1 axis regulates cell viability under an oxidative status (Figures 7(a) and 7(b)). Cells received exposure to 100 *μ*M H_2_O_2_ for 0.5 h to induce oxidative stress, and we noticed more retention of cell viability in groups treated with 200 nM and 1000 nM rapamycin while we observed a cell viability decline when cells were treated with 1 mM and 8 mM 3-MA (Figure 7(a)). Moreover, miR-27a mimic and si-FOXO1 transfection also reduced the cell viability in oxidative circumstance (Figure 7(b)). It is noticeable that rapamycin enhanced the cell viability in the NC group but with a lesser extent in the miR-27a and si-FOXO1 group. Together, these results indicated that the inhibition of autophagy by miR-27a-FOXO1 axis caused a cell viability decrease when they are exposed on H_2_O_2_, while the promotion of autophagy by rapamycin partially rescued this H_2_O_2_-induced cell death if the miR-27a-FOXO1 axis is not extremely aberrant activated. To take a further step in unraveling the mechanism of miR-27a-correlated cell death, we measured the ROS levels in ARPE-19 cells. As shown in Figure 7(c), ARPE-19 cells pretreated with rapamycin had a lower fluorescence intensity while cells pretreated with 3-MA had a higher fluorescence intensity. Our results confirmed that promotion of autophagy in ARPE-19 cells could significantly reduce the cytosolic ROS level. Next, we questioned whether the aberrant activation of miR-27a/FOXO1 axis could promote ROS accumulation in ARPE-19 cells. We used a confocal microscope to monitor the ROS level in a time-dependent manner.  Figure 7(d) showed that the fluorescence of ROS occurred earlier in the miR-27a and si-FOXO1 transfected group, which means higher cytosolic ROS levels, while cells transfected with miR-27a inhibitor had a slower fluorescence augmentation. Taken together, our results demonstrate that the miR-27a/FOXO1 axis could influence the ROS level in ARPE-19 cells through regulating autophagic activity. Next, we used flow cytometry to quantitatively analyze ROS production. Rapamycin treatment reduced ROS accumulation effectively, while 3-MA treatment promoted ROS accumulation slightly although the difference is not statistically significant (Figure 7(e)). Our results also confirmed that miR-27a and si-FOXO1 transfection induced ROS accumulation through repressing autophagy (Figure 7(f)).

In conclusion, H_2_O_2_ induces the overexpression of miR-27a and inhibits FOXO1 expression. The abnormal activation of miR-27a-FOXO1 axis inhibits the autophagy of ARPE-19 cells and promotes ROS accumulation, further forming a feedback loop and causing cell death.

## 4. Discussion

Since AMD is an age-related disease, the accumulation of toxic products and RPE function declining with age are two causal factors for the disease. Previous studies have showed that the primary changes in AMD are mainly the decline and disintegration of the RPE layer due to the degeneration of RPE cells [18]. Because aging is a chronic process, thus, we speculated that there might be some gradually augmented elements that contribute to RPE degeneration, such as ROS. Our previous study showed that miR-27a, miR-29b, and miR-195-5p are overexpressed in AMD patients; among them, miR-27a was identified as an oxidative stress-associated miRNA. Since RPE is under high oxidative circumstance, it is of great significance to establish an oxidative model to study the relationship between miR-27a and prooxidants in RPE cells.

Regarding the *in vivo* animal model, light-induced RD in mice has been proven to be a successful AMD animal model that is partially associated with oxidative stress [19]. Exposure to blue 460 nm wavelength LED is well known to lead to retinal histopathological and functional damage in rats [20], causing more intense RD than other LED exposure [21, 22]. We demonstrated a significant decrease in the ERG a- and b-wave amplitudes, massive photoreceptor cell death, and reduced thickness of the ONL in our blue-LED RD rat model.

Regarding the *in vitro* cell model, several studies have shown that different oxidative substances can stimulate cells to produce oxidative stress injury [23, 24]. H_2_O_2_ is a general substance to stimulate RPE cells to establish an *in vitro* cell oxidative stress model [6, 25]. Our results showed that H_2_O_2_ treatment successfully induced oxidative cell model as we could detect moderate decline of cell viability and ROS accumulation in ARPE-19 cells.

The interplay of genetic and environmental factors confers the complexity of pathogenesis of AMD [26]. Researches have shown that miRNAs play an essential role in retinal biogenesis [27]. It is noticeable that circulating miRNAs, which are found in peripheral blood, have been shown to participate in AMD progress through modulating immune response, oxidant-mediated cell injury, and angiogenesis [28]. Zhou et al. demonstrated that inhibition of miR-27a and miR-23a may serve to repress angiogenesis both in vivo and in vitro [29]. Lin et al. showed that the macular RPE cells of AMD patients had lower miR-23a expression level compared with that of controls and miR-23a might also play an antioxidative role in the retina [30]. Others demonstrated that ischemia-induced retinal neovascularization (NV) of rats was significantly reduced by intraocular injection of pre-miR-184, pre-miR-150, and pre-miR-31, which indicated the potential therapeutic role of miRNAs in retinal disease [31]. Therefore, we here questioned that whether our previously detected abnormally expressed miRNAs can participate in the development of AMD. Surprisingly, in our oxidative model, we detected an upregulation of miR-27a. This result strongly indicated that miR-27a might play some role in oxidant-mediated RPE injury. Moreover, we may find an explanation to the overexpression of miR-27a in the blood of AMD patients; that is, long-term light exposure and oxidative stress promotes RPE cells to express more miR-27a and to transport to blood.

To further explore the effects of miR-27a, we have to assess whether miR-27a exerts its function by inhibiting expression of target genes. As previously mentioned, FOXO1 was confirmed to have the opposite expression situation to miR-27a when under oxidative stress. So, luciferase reporter assay, western blot, and qRT-PCR were conducted and we showed that miR-27a directly targets 3′UTR of FOXO1 mRNA and regulates the mRNA and protein expression of FOXO1. From a pathophysiological point of view, researchers have indicated that excessive ROS or inadequate defense mechanisms of oxidative stress *in vivo* can cause multiple molecular damages, including hydroxylation of structural proteins and enzyme proteins, oxidation of ROS, and lipid peroxidation [32]. Autophagy is one of the main ways to degrade cell components in eukaryotic cells. It was often correlated with cell death since previous studies observed the accumulation of autophagosomes in dying cells. However, it is difficult to designate the specific roles of autophagy in regulating cell death because it is highly dependent on the specific circumstances [33]. Besides being associated with cell death, autophagy can also clear organs and protein aggregates at the basic level and reduce ROS accumulation and RPE dysfunction [34]. There is a balance between ROS generation and elimination in cells. The aging-associated decline of autophagic activity was reported to devastate this balance and lead to age-related diseases, including AMD [35, 36]. Mitter and colleague had proved inhibition of autophagy by 3-MA or by knockdown of ATG7 or BECH1 increased ROS generation and reduced cell viability [37]. As a key transcription factor, substantial evidences indicate that FOXO1's function relies on the modulation of downstream targets such as autophagy-associated genes and antioxidative enzymes [17]. Here, we tried to elaborate the effects of miR-27a-FOXO1 axis on autophagy and the subsequent influences. Our results proved that retinal damage induced by blue LED was aggravated by FOXO1 gene knockdown or miR-27a overexpression, while the damage was alleviated by FOXO1 gene overexpression or miR-27a inhibition. In vitro results demonstrated that overexpression of miR-27a reduced autophagy level of ARPE-19 cells under oxidative stress condition, then results in ROS accumulation and cell viability decline. Of note, pretreatment of autophagy enhancer rapamycin could only promote cell viability to a moderate extent in ARPE-19 cells transfected with miR-27a and si-FOXO1. This indicated that miR-27a-FOXO1 axis is essential in mediating the protective effects of autophagy. According to our present results, we speculate that long-term light exposure causes ROS accumulation in the retina; then, miR-27a is upregulated as a response to aggravated oxidation. Subsequently, overexpression of miR-27a inhibits the FOXO1-related autophagic function of RPE cells. The inhibition of autophagy accelerates ROS aggregation and furtherly promotes miR-27a expression. The feedback loop leads to an imbalance of ROS production and autophagy, causing RPE cell death and retinal degeneration. It will be immensely significant if further studies can verify the abnormal activity of miR-27a-FOXO1 axis in the retina of AMD patients. In conclusion, we demonstrated a novel role of miRNA-27a-FOXO1 axis in oxidation-induced RPE cell death. These findings may provide further evidence for the pathogenesis of retinal degenerative disorders such as AMD.

## 5. Conclusion

The results suggest that miR-27a is a negative regulator of FOXO1. Also, our data support the pivotal role of miR-27a-FOXO1 axis in modulating ROS-mediated retinal injury and RPE cell death. In conclusion, our findings explain the age-related ROS accumulation and RPE cell death from a novel perspective. These findings may provide some hints for future researches in AMD.

## Data Availability

The data used to support the findings of this study are included within the article.

## References

[1] Kaarniranta K., Salminen A. (2009). Age-related macular degeneration: activation of innate immunity system via pattern recognition receptors. *Journal of Molecular Medicine (Berlin, Germany)*.

[2] Resnikoff S., Pascolini D., Etya'ale D. (2004). Global data on visual impairment in the year 2002. *Bulletin of the World Health Organization*.

[3] Handa J. T., Bowes Rickman C., Dick A. D. (2019). A systems biology approach towards understanding and treating non- neovascular age-related macular degeneration. *Nature Communications*.

[4] Rodrigues G. (2014). A spontaneous CNV model provides new tool to understand AMD. *Investigative Ophthalmology & Visual Science*.

[5] Jarrett S. G., Boulton M. E. (2012). Consequences of oxidative stress in age-related macular degeneration. *Molecular Aspects of Medicine*.

[6] Datta S., Cano M., Ebrahimi K., Wang L., Handa J. T. (2017). The impact of oxidative stress and inflammation on RPE degeneration in non- neovascular AMD. *Progress in Retinal and Eye Research*.

[7] Kim S. Y., Kambhampati S. P., Bhutto I. A., McLeod D. S., Lutty G. A., Kannan R. M. (2021). Evolution of oxidative stress, inflammation and neovascularization in the choroid and retina in a subretinal lipid induced age-related macular degeneration model. *Experimental Eye Research*.

[8] Vasudevan S., Tong Y., Steitz J. A. (2007). Switching from repression to activation: microRNAs can up-regulate translation. *Science*.

[9] Cheng Y., Liu X., Zhang S., Lin Y., Yang J., Zhang C. (2009). MicroRNA-21 protects against the H_2_O_2_-induced injury on cardiac myocytes via its target gene PDCD4. *Journal of Molecular and Cellular Cardiology*.

[10] O'Day E., Lal A. (2010). MicroRNAs and their target gene networks in breast cancer. *Breast Cancer Research*.

[11] Ren C., Liu Q., Wei Q. (2017). Circulating miRNAs as potential biomarkers of age-related macular degeneration. *Cellular Physiology and Biochemistry*.

[12] Zhao Y., Dong D., Reece E. A., Wang A. R., Yang P. (2018). Oxidative stress-induced miR-27a targets the redox gene nuclear factor erythroid 2-related factor 2 in diabetic embryopathy. *American Journal of Obstetrics and Gynecology*.

[13] Deng Q., Dai X., Guo H. (2014). Polycyclic aromatic hydrocarbons-associated microRNAs and their interactions with the environment: influences on oxidative DNA damage and lipid peroxidation in coke oven workers. *Environmental Science & Technology*.

[14] Lei H., Quelle F. W. (2009). FOXO transcription factors enforce cell cycle checkpoints and promote survival of hematopoietic cells after DNA damage. *Molecular Cancer Research*.

[15] Martins R., Lithgow G. J., Link W. (2016). Long live FOXO: unraveling the role of FOXO proteins in aging and longevity. *Aging Cell*.

[16] Yang P., Peairs J. J., Tano R., Jaffe G. J. (2006). Oxidant-mediated Akt activation in human RPE cells. *Investigative Ophthalmology & Visual Science*.

[17] Xing Y. Q., Li A., Yang Y., Li X. X., Zhang L. N., Guo H. C. (2018). The regulation of FOXO1 and its role in disease progression. *Life Sciences*.

[18] van Lookeren Campagne M., LeCouter J., Yaspan B. L., Ye W. (2014). Mechanisms of age-related macular degeneration and therapeutic opportunities. *The Journal of Pathology*.

[19] Wei Q., Liang X., Peng Y. (2018). 17beta-estradiol ameliorates oxidative stress and blue light-emitting diode-induced retinal degeneration by decreasing apoptosis and enhancing autophagy. *Drug Design, Development and Therapy*.

[20] Kim G. H., Kim H. I., Paik S. S., Jung S. W., Kang S., Kim I. B. (2016). Functional and morphological evaluation of blue light-emitting diode-induced retinal degeneration in mice. *Graefe's Archive for Clinical and Experimental Ophthalmology*.

[21] Kuse Y., Ogawa K., Tsuruma K., Shimazawa M., Hara H. (2014). Damage of photoreceptor-derived cells in culture induced by light emitting diode-derived blue light. *Scientific Reports*.

[22] Shang Y. M., Wang G. S., Sliney D., Yang C. H., Lee L. L. (2014). White light-emitting diodes (LEDs) at domestic lighting levels and retinal injury in a rat model. *Environmental Health Perspectives*.

[23] Huang B., Fu H., Yang M., Fang F., Kuang F., Xu F. (2009). Neuropeptide substance P attenuates hyperoxia-induced oxidative stress injury in type II alveolar epithelial cells via suppressing the activation of JNK pathway. *Lung*.

[24] Yao L. L., Wang J. N., Huang Y. Z., Guo L. Y., Kong X. (2006). The protective effect of PEP-1-CAT fusion protein on hydrogen peroxide-induced oxidative stress injury in human umbilical vein endothelial cells. *Zhonghua Xin Xue Guan Bing Za Zhi*.

[25] Gong C., Qiao L., Feng R. (2020). IL-6-induced acetylation of E2F1 aggravates oxidative damage of retinal pigment epithelial cell line. *Experimental Eye Research*.

[26] Arora A., McKay G. J., Simpson D. A. (2007). Prediction and verification of miRNA expression in human and rat retinas. *Investigative Ophthalmology & Visual Science*.

[27] Wang S., Koster K. M., He Y., Zhou Q. (2012). miRNAs as potential therapeutic targets for age-related macular degeneration. *Future Medicinal Chemistry*.

[28] Su Y., Yi Y., Li L., Chen C. (2021). circRNA-miRNA-mRNA network in age-related macular degeneration: from construction to identification. *Experimental Eye Research*.

[29] Zhou Q., Gallagher R., Ufret-Vincenty R., Li X., Olson E. N., Wang S. (2011). Regulation of angiogenesis and choroidal neovascularization by members of microRNA-23~27~24 clusters. *Proceedings of the National Academy of Sciences of the United States of America*.

[30] Lin H., Qian J., Castillo A. C. (2011). Effect of miR-23 on oxidant-induced injury in human retinal pigment epithelial cells. *Investigative Ophthalmology & Visual Science*.

[31] Shen J., Yang X., Xie B. (2008). MicroRNAs regulate ocular neovascularization. *Molecular Therapy*.

[32] Da Costa L. A., Badawi A., El-Sohemy A. (2012). Nutrigenetics and modulation of oxidative stress. *Annals of Nutrition & Metabolism*.

[33] Denton D., Kumar S. (2019). Autophagy-dependent cell death. *Cell Death and Differentiation*.

[34] Lee J., Giordano S., Zhang J. (2012). Autophagy, mitochondria and oxidative stress: cross-talk and redox signalling. *The Biochemical Journal*.

[35] Cuervo A. M. (2008). Autophagy and aging: keeping that old broom working. *Trends in Genetics*.

[36] Kaarniranta K., Sinha D., Blasiak J. (2013). Autophagy and heterophagy dysregulation leads to retinal pigment epithelium dysfunction and development of age-related macular degeneration. *Autophagy*.

[37] Mitter S. K., Song C., Qi X. (2014). Dysregulated autophagy in the RPE is associated with increased susceptibility to oxidative stress and AMD. *Autophagy*.

